# Questions Prepared and Adopted by the State Boards of Dental Examiners

**Published:** 1883-09

**Authors:** 


[A general wish was expressed by those present that the following list be published in connection with these proceedings, as giving some idea of the scope and range of the examinations contemplated.—ED. REGISTER.]

### Questions Prepared and Adopted at a Meeting of the Representatives of the Various State Boards of Dental Examiners,

*Held at Lexington, Ky., Feb. 22d, 1883, for use by those acting under* LAWS REGULATING THE PRACTICE OF DENTISTRY.

---

### ANATOMY.

1. Name five of the principle tissues of the body.
2. Of what does the skeleton consist?
3. What is the immediate covering of the bones?
4. Name, locate, and describe the muscles of mastication.
5. Name the bones of the face.
6. Locate and describe the Maxillary sinus.
7. Name, locate, and describe the Salivary Glands.
8. Give the origin and course of the nerves which supply the teeth.
9. Name the different parts of the vascular system.
10. Name two of the largest glands of the body.
11 Name the muscles which depress the lower jaw.
12. Name the facial muscles, giving their origin and insertion.

### PHYSIOLOGY.

1. Describe the process of digestion.
2. Name and describe the digestive secretions.
3. What is the function of the liver, of the kidneys, and of the skin?
4. Describe the circulation of the blood and the physiological action produced by it.

5. Describe some of the changes produced in the air taken into the lungs by the process of respiration.

6. Give some of the sources of the carbonic acid exhaled from the lungs.

7. Name some of the chief functions of the nervous system.

8. What is reflex-nervous action? Give example.

9. What cranial nerve being a motor nerve at its origin joins a nerve of sensation and becomes a nerve of special sense?

10. What reciprocal influences do the sensory and sympathetic nerves have upon each other?

### HISTOLOGY.

1. From what class of tissues do tooth-germs originate?
2. Give the minute anatomy of the three hard structures of the tooth and the order of their development.
3. Name and describe the structure of muscular fiber.
4. Describe the corpuscular elements of the blood.
5. Name and describe the layers of the mucous membrane.
6. Describe the nerve tissues.
7. Describe the formation and structure of bone.

### PATHOLOGY.

1. What is disease?
2. Define the various stages of inflammation.
3. Explain the difference between caries and necrosis of bone.
4. Describe exostosis. What induces it?
5. What is the difference between fever and inflammation?
6. In what ways may inflammation terminate?
7. Describe the source and formation of pus.
8. Give the distinctive features of Neuralgia.
9. What conditions of tooth-pulp occasion pain?
10. Name the agents concerned in, and describe the process of dental caries.
11. Name and describe the common diseases of the gum.

### MATERIA MEDICA.

1. From what sources are medicinal agents obtained?
2. Name six of the remedial agents derived from the vegetable kingdom.

3. Name six obtained from the mineral kingdom.

4. From what other sources are remedial agents or influences derived?

5. From what sources are the principal sedatives derived?

6. Name five valuable stimulants.

7. Name three efficient arterial sedatives.

8. Name and describe the action of two principal escharotics?

9. Name five important tonics.

10. Name in order of potency five of the leading poisons.

SURGERY.

1. What conditions demand surgical interference?

2. For what purpose or purposes are surgical operations performed?

3. What are some of the dangers to be feared from surgical operations?

4. What are the means employed for arresting excessive hemorrhage?

5. Describe your method of opening the Maxillary sinus for treatment.

6. What operations are resorted to in treatment of Neuralgia?

7. Describe your treatment for fracture of the Inferior Maxilla, between the cuspid and bi-cuspid tooth.

8. How would you proceed to reduce a partial or complete dislocation of the lower jaw?

9. Describe the operation for Ranula.

10. What conditions of the Maxillary sinus require surgical interference?

THERAPEUTICS.

1. What is Therapeusis?

2. To what does prophylaxis refer?

3. What do you understand by abortive measures?

4. What is the object of palliative treatment?

5. What are the principles involved in the treatment of inflammation?

6. What are the points of treatment for Alveolar Abscess?

7. In what cases may diseased tooth pulp be restored and preserved, and by what treatment?

8. Give treatment for simple inflammation of the gums.

9. Give the treatment for Aphthous sore mouth, and Pyorrhea Alveolaris.

10 Give a list of therapeutic agents valuable to the dentist in general practice.

### CHEMISTRY.

1. What is an element?

2. How many elements are there?

3. What is a compound substance?

4. In what conditions are elements found in nature?

5. What properties are possessed in common by nearly all the elements.

6. What element is found most abundant in nature?

7. For what element is there the most extensive affinity?

8. What classes of substances are the best conductors of heat and cold?

9. What are the chemical constituents of Dentine and Enamel, and what are the proportions of each?

10. What are the chemical constituents of Saliva?

### OPERATIVE DENTISTRY.

1. What is Dental Periostitis, in what respect is it peculiar, and what is the treatment?

2. Name and describe the various affections of tooth pulp and the treatment proper for each.

3. Give the pecularities of hyper-sensitiveness of dentine, with principles and modes of treatment.

4. Describe the common points to be observed in the preparation of a cavity for filling.

5. Name and describe the various steps in the introduction of a gold filling.

6. Mention some of the common causes of failures in filling teeth.

7. How does filling arrest the decay of the teeth?

8. What qualities are requisite in materials used for filling teeth?

9. Give the origin, manner of deposit, and effects of salivary calculus.

### PROSTHETIC DENTISTRY.

1. What anatomical and physiological changes are occasioned by the loss of the teeth?

2. What restoration is Prosthetic Dentistry able to give the toothless?

3. Name in the order of their importance, five points that should be attained in the insertion of artificial teeth.

4. What are the requisite properties of materials for constructing artificial dentures. Name the different materials in use, in the order of their value?

5. In what condition should the mouth be for the proper reception of a denture?

6. Describe a method or methods of making an accurate model for constructing a set of teeth.

7. What will guide in the arrangement of artificial teeth?

8. Describe the various modes of retaining artificial teeth in the mouth.

9. What are the advantages and disadvantages of Gold, of Continuous Gum, of Rubber, and of Celluloid as a base?

### IRREGULARITIES.

1. What are the most common causes of irregularity of the teeth?

2. Give instances of irregularities of a congenital nature, with approved modes of treatment.

3. Name some accidental irregularities and manner of treating them.

4. What diseases are most frequent causes of lesions of the jaws and palate?

5. Under what circumstances will neglect or unwise treatment of the temporary teeth lead to imperfect development of the permanent ones?

6. Are the Maxillary arches capable of expansion--if so, by what means?

7. What would be the best means of regulating a crowded arch with the cuspids standing inside the line?

